# The Development of an Effective Bacterial Single-Cell Lysis Method Suitable for Whole Genome Amplification in Microfluidic Platforms

**DOI:** 10.3390/mi9080367

**Published:** 2018-07-25

**Authors:** Yuguang Liu, Dirk Schulze-Makuch, Jean-Pierre de Vera, Charles Cockell, Thomas Leya, Mickael Baqué, Marina Walther-Antonio

**Affiliations:** 1 Department of Surgery, Division of Surgical Research, Mayo Clinic, Rochester, MN 55905, USA; liu.yuguang@mayo.edu; 2 Astrobiology Group, Center of Astronomy and Astrophysics, Technical University, 10623 Berlin, Germany; schulze-makuch@tu-berlin.de; 3 German Aerospace Center (DLR), Institute of Planetary Research, Management and Infrastructure, Astrobiological Laboratories, 12489 Berlin, Germany; jean-pierre.devera@dlr.de (J.-P.d.V.); Mickael.Baque@dlr.de (M.B.); 4 School of Physics and Astronomy, University of Edinburgh, Edinburgh EH9 3FD, UK; c.s.cockell@ed.ac.uk; 5 Fraunhofer Institute for Cell Therapy and Immunology, Branch Bioanalytics and Bioprocesses (IZI-BB), Extremophile Research & Biobank CCCryo, 14476 Potsdam, Germany; Thomas.Leya@izi-bb.fraunhofer.de; 6 Department of Obstetrics and Gynecology, Mayo Clinic, Rochester, MN 55905, USA

**Keywords:** bacteria lysis protocol, microalgae lysis, single-cell multiple displacement amplification

## Abstract

Single-cell sequencing is a powerful technology that provides the capability of analyzing a single cell within a population. This technology is mostly coupled with microfluidic systems for controlled cell manipulation and precise fluid handling to shed light on the genomes of a wide range of cells. So far, single-cell sequencing has been focused mostly on human cells due to the ease of lysing the cells for genome amplification. The major challenges that bacterial species pose to genome amplification from single cells include the rigid bacterial cell walls and the need for an effective lysis protocol compatible with microfluidic platforms. In this work, we present a lysis protocol that can be used to extract genomic DNA from both gram-positive and gram-negative species without interfering with the amplification chemistry. *Corynebacterium glutamicum* was chosen as a typical gram-positive model and *Nostoc* sp. as a gram-negative model due to major challenges reported in previous studies. Our protocol is based on thermal and chemical lysis. We consider 80% of single-cell replicates that lead to >5 ng DNA after amplification as successful attempts. The protocol was directly applied to *Gloeocapsa* sp. and the single cells of the eukaryotic *Sphaerocystis* sp. and achieved a 100% success rate.

## 1. Introduction

Phenotypically identical cells from the same population can have dramatic heterogeneity in their behavior. This heterogeneity plays a significant role in various biological processes including tumor progression [1,2] and immune response [3]. Efforts have been focused on exploring the heterogeneous behavior within the same population such as cell growth [4] and drug responses [5,6,7,8] using novel molecular reporters and advanced imaging tools. These technologies are effective and popular, however, to better understand the reason behind the different behaviors, it is necessary to identify the variance in the genomes of these genetically similar cells on a single-cell level.

Single-cell whole genome sequencing (SC-WGS) is emerging as a promising tool for investigating the genetic diversity and heterogeneity of complex biological systems [9,10,11]. This technology offers the ability of identifying genome mutations of a single cell within a population, which is often neglected in standard studies such as metagenomics [12,13]. As a result, SC-WGS is starting to impact our understanding of human physiology and diseases and bring forth new prospects such as evaluating the role of genetic mosaicism [14] in diseases like cancer and facilitating the analyses of uncultured species in environmental studies [15,16].

The key steps in SC-WGS include single-cell isolation, lysis and amplifying genomic DNA from femto to picograms to reach the quantity sufficient for standard library preparation and sequencing (>25 ng) [17,18]. Microfluidic platforms are often used for amplifying genome from single cells due to their unique ability of handling nanoliters of fluid in a controlled manner [19,20,21,22,23,24,25], thus, allowing for the isolation of single cells into compartments for lysis and genome amplification [26,27]. Multiple displacement amplification (MDA) [28] has been a popular option for single-cell whole genome amplification (SC-WGA) in microfluidic platforms [29,30,31]. It is based on φ29 DNA polymerase and random primers to replicate template DNA with high fidelity and lower error rates following relatively simple procedures compatible with microfluidic systems [32,33,34]. 

So far, most of the SC-WGS applications have been focused on human cells that can be easily lysed using commercial SC-WGA kits. SC-WGS of bacterial species is still rare due to the 1000× lower starting genomic DNA and the rigid and multi-layered cell walls of these microorganisms [35]. The cell wall of gram-positive bacterial species generally consists of a thick layer of peptidoglycan outside of the cytoplasmic cell membrane and, thus, is harder to penetrate. The cell wall of gram-negative bacterial species generally has a thinner peptidoglycan layer between its outer and inner lipid membrane, and are relatively easier to lyse. However, some gram-negative species have much thicker and complex cell walls than most gram-negative microbes and are particularly resilient. For example, the cell wall of cyanobacteria species contains a thick and highly cross-linked peptidoglycan layer and a surface layer composed of polymerized proteins and exopolysaccharide [36], making it especially difficult to penetrate.

Many standard bacterium lysis methodologies efficient for bulk studies such as bead beating and high pressure are not applicable to microfluidic platforms [37,38,39]. Several existing methods effective for the lysis of rigid species such as cyanobacterium were based on mechanical disruptions including sonication and lyophilization [40], but these are low-throughput and not suitable for single-cell studies in microfluidic systems. Others reported genetic-based methods which include expressing a lytic cassette from a bacteriophage in certain strains [41], but these rely on labor-intensive strain-specific genetic engineering and are not generally adaptable. 

Chemical-based bacterial lysis is a popular option in microfluidic-based SC-WGA. However, the key challenge is to use chemicals with minimal interference with the subsequent MDA amplification chemistry. Due to the challenge of implementing wash steps in the microfluidic-based WGA process, not all chemicals common for effective bacterial cell lysis are applicable. For example, ionic surfactants such as sodium dodecyl sulfate and sarkosyl are often avoided in microfluidic-based WGA because they almost completely inhibit polymerase activities [42]. Chemicals including phenol and spermine have been proven effective for degrading bacterial cell walls, however, due to the toxicity, a fume hood is required, thus, making it difficult to use in microfluidic systems. Freeze-thaw combined with alkaline treatment has been proven highly efficient for the lysis of bacterial single cells for SC-WGA in well plates [43], but requires placing the plates in −80 °C freezer for 1 h, therefore it is not applicable for microfluidic experimental setups with complex controls. 

To overcome these hurdles, others have pursued bacterial SC-WGA in microfluidic chips by pre-lysing the cell population off-chip using ionic surfactants followed by standard in-tube wash prior to introducing them into the chips [42]. This attempt has led to satisfactory results for studying pure cultures cultivated in laboratory settings with an initial on-chip amplification followed by a second round of amplification off-chip in a standard manner. Nevertheless, the off-chip pretreatments are not suitable for bacterial cells in low abundance or are in a complex community such as clinical or environmental samples. Besides, the pre-lyse step would possibly introduce extracellular contaminant DNA when amplifying the extremely low amount of DNA from a single bacterial cell. To perform the entire process on-chip, multiple rounds of amplification can be an alternative to counter the insufficient cell lysis, but it increases the percentage of duplicated reads and thus bias in the data analyses. 

To extend the use of microfluidic platforms for SC-WGS to microbiological and microbiome research, it is essential to develop protocols suitable for on-chip lysis of a single bacterial cell that circumvents the aforementioned concerns. Moreover, cell wall structures of different bacterial species vary, hence it would be ideal for a protocol to be easily adapted to various species without extensive efforts. Therefore, in this work, we develop a bacterial single-cell lysis protocol as a guideline for MDA-based bacterial SC-WGA in microfluidic platforms that produces >25 ng of genomic DNA per cell, sufficient for downstream library preparation. This on-chip protocol combines three primary bacterial lysis methods which include thermal [15,43,44], enzymatic [45,46] and chemical lysis [47,48,49] and was tested on both gram-positive and gram-negative bacterial species for subsequent on-chip SC-WGA. In this study, *Corynebacterium glutamicum* was used as a typical gram-positive model whose cell wall is thicker than most gram-negative species. Besides, Corynebacterium species are increasingly recognized as the occasional causes of prosthetic joint infection associated with significant morbidity [50]; and this disease has a low organism burden and usually involves infection often caused by commensal flora, and thus requiring higher sensitivity and specificity for its identification [51]. *Nostoc* sp. was chosen as a gram-negative model due to the significant lysis difficulties encountered in previous studies [36,52]. The developed protocol was then tested on *Gloeocapsa* sp. and *Sphaerocystis* sp. due to the significant lysis difficulty and the viscous extracellular matrix that largely hinders chemical penetration, and a 100% success was achieved for both species. In addition, *Nostoc* sp. and *Gloeocapsa* sp. belong to the cyanobacteria, and cf. *Sphaerocystis* sp. (hereafter referred to as *Sphaerocystis* sp.) is a genus of green algae (Chlorophyceae), and these species are of high interest in astrobiological and environmental studies as such taxa were responsible for creating the oxygen atmosphere through photosynthetic activities billions of years ago. We believe that the effective on-chip lysis method that enables successful genome amplification of the chosen species would serve as a guideline for bacterial single-cell genomics in microfluidic platforms, and can be applied to a wide range of applications including biomedical research, environmental studies, and future human space exploration missions. 

## 2. Materials and Methods

### 2.1. Cell Wall Components of Chosen Bacterial Cells

**Figure 1:**
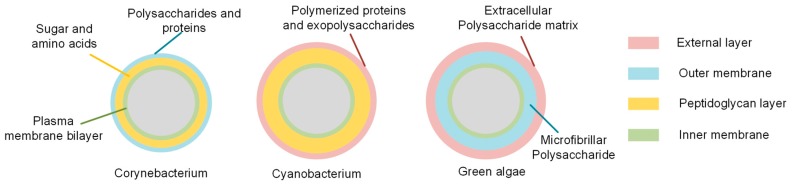
A representative illustration of the cell envelops of Corynebacterium, cyanobacterium species, and green algae.

The components of the cell wall are illustrated in Figure 1. Generally, the envelope of *Corynebacterium* spp. consists of an outer membrane primarily composed of polysaccharides and proteins, a cell wall of peptidoglycan layers and a typical plasma membrane bilayer as the inner membrane [53]; while the envelope of cyanobacterial species mainly consists of an external layer composed of exopolysaccharide and polymerized proteins, an outer membrane, a much thicker peptidoglycan layer and an inner cytoplasmic membrane [54]. *Sphaerocystis* sp. is a genus of green algae (Chlorophyta), in which the cell wall surrounds the cytoplasm membrane and usually is composed of microfibrillar polysaccharides and is covered by an extracellular polysaccharide matrix [55,56]. Therefore, the lysis protocol was designed to sequentially break through the cell envelope from the outermost to the innermost layer with minimal interference with φ29 DNA polymerase activity.

Others have proven that following the lysis instruction of the Relpli-g Single Cell kit (Qiagen) would achieve a 90% amplification rate of single *Escherichia coli* [27]. However, only 30% of *C. glutamicum* single cells were amplified with an average of 15.78 ng DNA in the same manner. This is likely due to the fact that the peptidoglycan layer of gram-positive species is multilayered, with a thickness range of 30–100 nm, while the gram-negative species has a single-layered peptidoglycan layer of 2–10 nm [54,57]. This shows that additional treatments are necessary to sufficiently lyse species with thicker cell walls. 

### 2.2. Cell Preparation

*C. glutamicum* (donated by Dr. Robin Patel, Mayo Clinic, Rochester, MN, USA) was cultured in a nutrient broth (DB) at 37 °C and harvested during log phase and diluted in a sample diluent (0.08% Pluronic F127 (Sigma Aldrich, St. Louis, MO, USA) in Phosphate Buffer Saline (PBS)) to ~10^6^/mL to facilitate single-cell trapping. The Antarctic strain CCCryo 231-06 of the cyanobacterium *Nostoc* sp. and the Arctic strain CCCryo 101-99 of cf. *Sphaerocystis* sp. (cf. = Latin.: confer, meaning “needs to be discussed”; the taxonomic identity of this strain is not yet fully resolved) were obtained from the Culture Collection of Cryophilic Algae (CCCryo) at the Branch Bioanalytics and Bioprocesses of the Fraunhofer Institute for Cell Therapy and Immunology (IZI-BB) in Potsdam. They were collected, cultured, and maintained in cooperation with the German Aerospace Center (DLR) Berlin. *Gloeocapsa* sp. was obtained from the University of Edinburgh, UK. All samples were received in the desiccated form and re-suspended in the sample diluent. 

### 2.3. Microfluidic Experimental Setup

**Figure 2:**
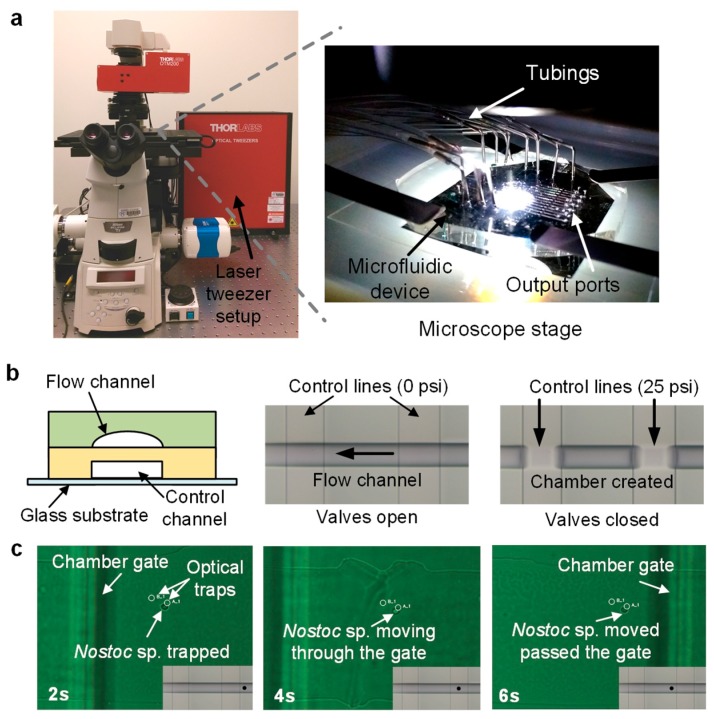
An overview of the optofluidic single-cell whole genome amplification (SC-WGA) platform. (**a**) This platform integrates an advanced microscope, optical tweezers, and a microfluidic device. The device consists of 12 parallel reaction systems. (**b**) The double-layer microfluidic device consists of flow channels in the top layer and control channels in the bottom layer. Chambers are formed by pressuring control channels into flow channels at their junctions. (**c**) Time-elapsed images of a single *Nostoc* sp. cell is trapped and moved into a chamber. The inset illustrates the location of the cell in the channel.

The study was performed in an optofluidic platform initially developed by Landry et al. [27] and reconstructed at the Mayo Clinic (Rochester, MN, USA). Briefly, this platform integrates a microscope (Nikon Eclipse, Melville, NY, USA), optical tweezers (1064 mn, Thorlabs, Newton, NJ, USA), and a customized Polydimethylsiloxane (PDMS) microfluidic chip with 12 parallel reaction systems (Figure 2a). Each reaction system has a series of valves that control the opening and closing of the chambers, allowing for the on-demand creation of isolated microenvironments (Figure 2b). The details of the device fabrication protocol are provided in the Supplementary Materials. Other microfluidic systems such as droplet microfluidics [58,59,60] are attractive alternatives due to their high-throughput, however, these are based-on random encapsulation based on probability which is more suitable for the studies of pure cultures. Due to the complexity of the *Nostoc* sp., *Gloeocapsa* sp. and *Sphaerocystis* sp. we obtained, optical tweezers were chosen to ensure higher target single-cell confidence with minimal contaminants.

In this study, the microfluidic channels for sample introduction were pre-soaked in the chip diluent (0.04% Pluronic F127 in PBS) for 30 min prior to experiments to prevent the cells from sticking to the PDMS channel surface during the cell sorting. Samples can be introduced into the chip, and single cells can be trapped and transported into microchambers by optical traps (Figure 2c). The valves of these chambers then can be closed to isolate the single cells. Visually identifiable contaminating cells can be trapped and transported out of the chambers to ensure only the target cell is in the chamber prior to the lysis step. The laser power at the objective was measured to be 50 mW, which was proven by others with marginal effects on cell viability [61,62]. Genomic mutation of bacterial cells under the effect of laser power has not been reported to the best of our knowledge, however, it would be valuable to characterize this aspect in our future single-cell genomic studies. Reagents include lysis buffers and DNA polymerase can be sequentially added to the isolated cells to perform chemical reactions. The amplified product can be collected from the outlet ports of the chip and transferred into microwell plates for downstream processing. All the supplies and reagents were filtered (0.2 µm), autoclaved or UV-sterilized, except for the DNA polymerase. Ten single-cell reactions and two negative control reactions were performed in each test.

### 2.4. Choice of Lysis Buffer Components

The study started with REPLI-g Single Cell Kit (Qiagen, Germantown, MD, USA) instruction as the baseline, along with the propriety D2 lysis buffer (concentrated alkaline) supplied in the kit. Due to the insufficiency of the standard alkaline-based lysis protocol, we hypothesized that the addition of brief heat-shock steps, an appropriate amount of lysozyme (Epicenter, Madison, WI, USA), Dithiothreitol (DTT) (Biorad, Hercules, CA, USA) and Ethylenediaminetetraacetic acid (EDTA) would significantly improve the lysis success for hard-to-lyse species according to the known effects of each of these chemicals on the bacterial cell wall [42,46,63]. Note that EDTA was reported for enabling the permeability of the outer membrane by chelating divalent cations that stabilizes negatively-charged sugars but with minor effects [36], therefore, we kept EDTA concentration at a constant value of 0.5 mM without further optimization. Details of the concentration of lysozyme and DTT used in the lysis buffer during the optimization process are presented in the Results and Discussion section. 

### 2.5. Microfluidic Bacterial Lysis for SC-WGA Workflow

**Figure 3:**
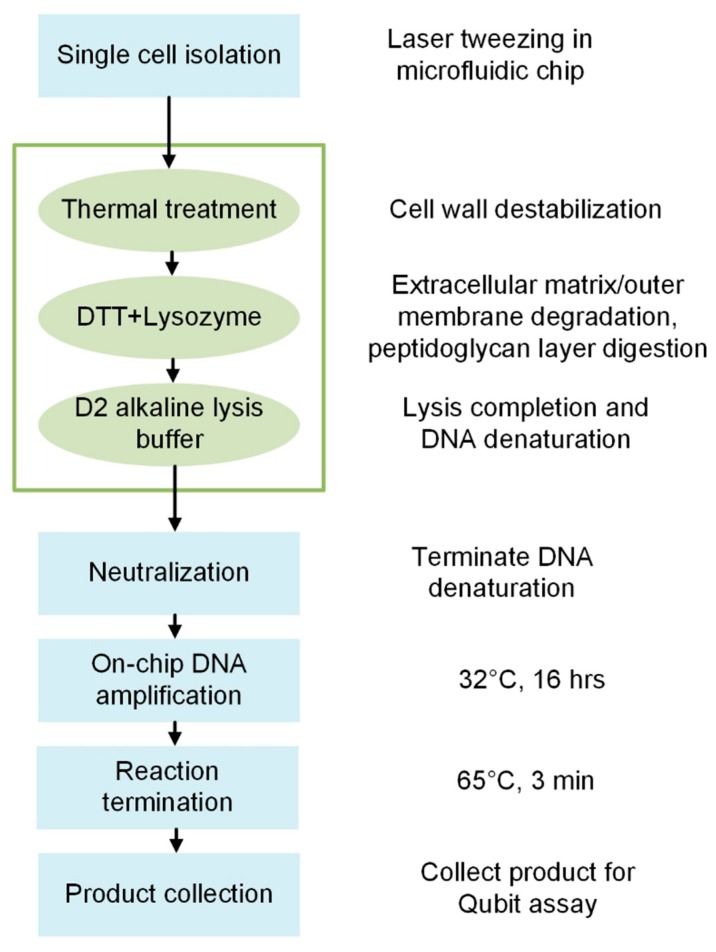
An overview of the workflow of single-cell isolation, lysis, and DNA amplification in a microfluidic chip. The blue rectangles represent standard steps in an on-chip SC-WGA process. The green box highlights the general steps of the lysis protocol developed and optimized.

The general workflow is shown in Figure 3. After single-cell isolation in the microfluidic chip, a heat-shock was performed by alternately placing the microfluidic chip on a hotplate and a cold block for a controlled amount of time. Lysis reagents were introduced sequentially and the microfluidic chip was placed on a hotplate for reagent incubation. After lytic reactions, the neutralized buffer in the kit was added to terminate DNA denaturation at room temperature. The polymerase was mixed according to the kit’s instruction and added into the reaction chambers, and the chip was placed on a hotplate at 32 °C for 16 h. The amplification reaction was terminated by incubating the microfluidic chip at 65 °C for 3 min and cooled on ice. Gel-loading pipette tips were inserted into the outlet ports of the chip, and nuclease-free water was introduced into the chip to flush the amplified product into the pipette tips until the fluid level reached the 20 µL mark. The product was collected and stored at 4 °C, and a high-sensitivity Qubit assay (Thermo Fisher, Waltham, MA, USA) was performed to assess the amount of the amplified genomic DNA from single cells. If the amplified genomic DNA from a single cell was >25 ng while the DNA is not detectable in negative controls, we considered it a successful lysis and amplification. No evaporation was observed during the process at elevated incubation temperatures (up to 95 °C) as the fluid was contained in closed chambers. Details of the lysis buffer incubation time and temperature used during the protocol optimization process are presented in the Results and Discussion section. Note that the results from the protocol development processes that did not lead to expected results (>25 ng) were also presented; however, we continued the optimization until the average amount of DNA from a single cell was amplified to >25 ng.

## 3. Results and Discussion

### 3.1. Optimization of Heat-Shock Treatment on C. glutamicum

**Figure 4:**
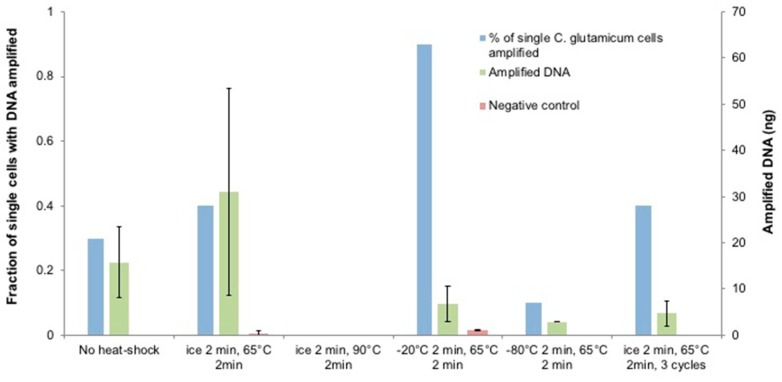
The temperature range effect of heat-shock on the *C. glutamicum* lysis for SC-WGA. After heat-shock treatment, the proprietary D2 alkaline lysis buffer was added and incubated at 65 °C for 10 min, according to the manufacturer’s instruction. *N* = 10 single cells per condition, error bars represent standard deviation.

Heat-shock is a physical treatment commonly used in bacterial transformation and lysis as it alters the fluidity of the cell membrane and creates pores due to the sudden change in temperature [63,64,65,66]. We tested the effects of heat-shock temperature range on *C. glutamicum* as a pretreatment prior to following the standard instruction (Figure 4). Performing heat-shock at different temperatures resulted in different single-cell amplification rates and yields. Ice/65 °C slightly increased the single-cell amplification rate by 10% and doubled the average amplified DNA amount, but the difference in amplified DNA amount between the two conditions is not statistically significant. However, the wide-spread amplified DNA amount (0.77–57.2 ng) suggests that the ice/65 °C treatment was sufficient for certain single-cell replicates, but had a minor effect on the others possibly due to the stochastic cell wall features of individual cells. To test if the heat-shock with a higher intensity would have a distinct effect on the amplification rate for *C. glutamicum* single cells, more aggressive heat-shock tests were attempted. When the upper temperature was increased to 90 °C, no detectable DNA was obtained after amplification. We assume that the amplification failure was caused by the over-denaturation of DNA rather than DNA degradation at high temperature, as DNA degradation starts at 100 °C [67] and generally its melting temperature lies between 50–100 °C depending on factors including genome size and guanine-cytosine content [68]. Since the D2 buffer already contained DNA denaturing agents, incubating it at elevated temperature could possibly accelerate DNA denaturation.

When the lower temperature was decreased to −80 °C, only 10% of the single cells amplified and resulted in 2.9 ng DNA, while −20 °C/65 °C led to 90% of single-cell amplification with 6.81 ng of DNA. Even though a more aggressive heat-shock is expected to exert stronger destabilization effect on the bacterial cell wall through mechanical fracturing, DNA shearing could happen during ice crystal formation [69] at extremely low temperatures which eventually lead to amplification failure. We further investigated the effect of the number of heat-shock cycles by repeating the test with three cycles of ice/65 °C treatment. This raised the lysis rate by 10% but lowered the amplified DNA to only a few nanograms, which suggests that the template DNA quality may have been compromised during the extended heat-shock cycles for *C. glutamicum*.

### 3.2. Optimization of Lysozyme Treatment on C. glutamicum

**Figure 5:**
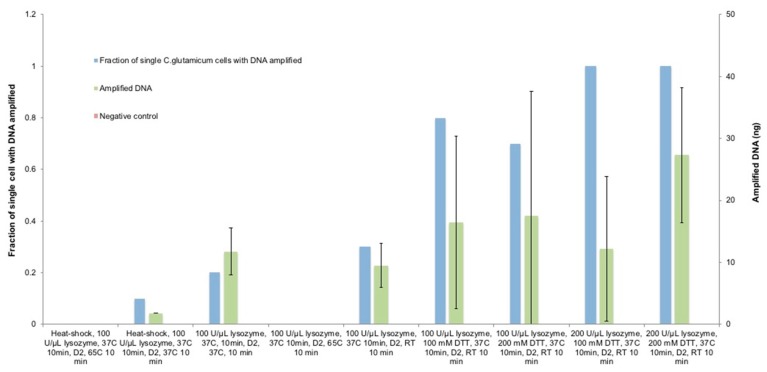
The optimization of the combined effect of thermal treatment, lysozyme, and DTT on *C. glutamicum* lysis to reach the optimal condition for subsequent SC-WGA. *N* = 10 single cells per condition, error bars represent standard deviation.

To further increase the lysis rate and amplification yield, lysozyme was used after the heat-shock due to its well-known effect of digesting the peptidoglycan layer [46,63]. We continued the lysis optimization based on the optimal lysis condition achieved so far with other variables unaltered (Figure 5). After one cycle of ice/65 °C heat-shock, 100 U/µL Ready-Lyse lysozyme was added and incubated at 37 °C for 10 min; after that, the D2 buffer was added and incubated at 65 °C for 10 min prior to stopping the lytic reaction. Surprisingly, no amplification was measured in any of the single-cell replicates. As a common bacterial DNA extraction reagent, lysozyme has not been reported to cause DNA degradation [46]; besides, 200 U/µL of lysozyme poses marginal deleterious effect on MDA-based genome amplification and has been widely used in various MDA reactions [42,58]. Due to these reasons, we assume that the released DNA was overly denatured during the incubation of D2 at 65 °C. We verified this assumption by lowering the D2 buffer incubation temperature to 37 °C, and this led to 10% of the amplification of single *C. glutamicum* cells with 1.76 ng DNA. However, the still poor performance suggests that the combined use of heat-shock, lysozyme, and alkaline reagents was too aggressive for *C. glutamicum* cells because the results above showed that the ice/65 °C treatment combined with the alkaline-based lysis was already sufficient for certain single-cell replicates. To find out an optimal condition, we used only lysozyme and the D2 buffer at 37 °C without heat-shock treatment, and this resulted in 20% of the single-cell genome amplification with an average of 11.72 ng DNA, and the single-cell amplification rate further increased to 30% with an average of 9.5 ng of DNA when the D2 buffer was incubated at room temperature, thus confirming our assumption. These results suggest that the elevated incubation temperature for the D2 alkaline buffer might have lysed the *C. glutamicum* cells more effectively but may have caused damages to the template DNA; moreover, this would narrow the window for finding out an ideal lysis condition while maintaining an acceptable DNA integrity for successful amplification. For this reason, we decided to proceed with the protocol optimization with the incubating D2 lysis buffer at room temperature rather than 37 °C since the results from these two conditions were not statistically different.

### 3.3. Optimization of Lysozyme Combined with DTT Treatment on C. glutamicum

Although using Ready-Lyse lysozyme followed by the D2 buffer did not lead to desirable improvements in single-cell amplification, we assume that the lysozyme failed to reach and digest the peptidoglycan layers in an effective manner. Therefore, the sulfhydryl compound DTT was used simultaneously with lysozyme because it is reported as an agent for degrading the external layers due to its ability of breaking disulfide bonds, disrupting polysaccharide, and altering the extracellular matrix of biofilms [70,71,72]. We used 100 mM DTT and 100 U/µL lysozyme simultaneously and incubated them at 37 °C for 10 min, followed by adding the D2 buffer and incubating it at room temperature for 10 min. Expectedly, the *C. glutamicum* single-cell amplification rate was raised to 80% with an average of 14.75 ng of DNA. However, under this lysis condition, most of the single-cell replicates amplified to only 6–17 ng, and merely one single cell amplified to 48 ng, thus leading to the widespread standard deviation. This shows that the same lysis condition could lead to significant differences in cell lysis due to the stochastic features of different single cells within the same species, which explains why it is generally easier to extract DNA from bulk cells without the need for intensive lysis protocol development. Comparing with previous conditions in Figure 5, the improvement of single-cell lysis and amplification by adding DTT implies that DTT assisted the bacterial cell lysis by permeating the external layers to enable lysozyme to degrade peptidoglycan layers. As an effort to further improve the amplification, the DTT concentration was increased to 200 mM while the lysozyme remained 100 U/µL, however, no significant improvement in the amplification rate was achieved but the DNA amount was increased by 21%, with the highest and lowest amplified DNA amount being 64 ng and 6 ng respectively. These results imply that 200 mM DTT is sufficient to break through the external layer for lysozyme to take effect. Further improvement of cell wall penetration would possibly rely on the increase of lysozyme concentration. 

**Figure 6:**
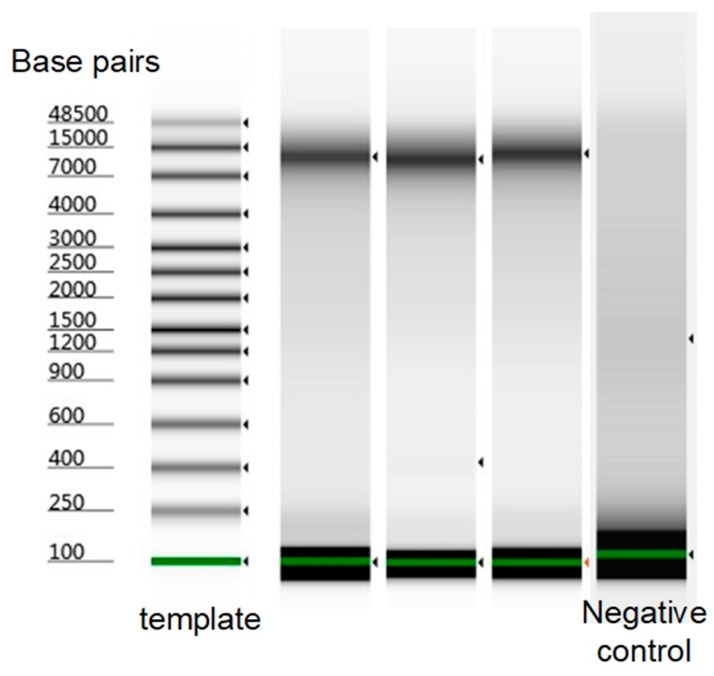
The tape station results of amplified DNA from single *C. glutamicum* cells using the optimized protocol. The DNA was not degraded. The results from three single *C. glutamicum* cells were shown, with one negative control.

To find out whether DTT or lysozyme was more significant to the lysis process, 100 mM DTT and 200 U/µL lysozyme was used instead, and this achieved a 100% single *C. glutamicum* amplification, however, the average DNA was lowered by 30%. These results imply that the increased concentration of lysozyme penetrated the outer membrane of all the *C. glutamicum* replicates in the presence of 100 mM DTT but with rather low speed. This can explain why only 2 replicates were amplified to >25 ng of DNA while the rest remained 6–10 ng. Thus, both DTT and lysozyme played a significant role and the decreased concentration of either would compromise the amplification. Therefore, as a final attempt, 200 mM DTT and 200 U/µL lysozyme was used at the same time; as predicted, this achieved a 100% amplification rate with an average of 29 ng DNA; and among the amplified single-cell replicates, 90% led to >25 ng DNA, improving significantly compared with the previous attempt (*p*-value = 0.017). After the expected success of lysis and amplification of single *C. glutamicum* was achieved, we assessed the integrity of the amplified DNA from single *C. glutamicum* cells in Tapestation (Agilent 2200, D1000 Screentape, Santa Clara, CA, USA), and the results show that the DNA was not degraded (Figure 6).

### 3.4. The Cyanobacteria Species’ Cell Wall Description

As a gram-negative species, cyanobacteria cell wall structure is perhaps the largest and the most diverse group of bacteria and is among the most challenging to break through. Some cyanobacteria can have peptidoglycan layers of over 700 nm [73], and the degree of cross-linking of peptidoglycan layer is 20–33% higher than most of the gram-negative species, similar to that of gram-positive species (56–63%) [74]. In addition, cyanobacterial outer membranes components include carotenoids and unusual fatty acids that are linked to the peptidoglycan layers via bridge-like coiled-coil domains [54]. These features make cyanobacterium perhaps the most difficult microbes to lyse for amplification and thus has been rarely studied on a single-cell level. Therefore, it is necessary to rely on the combined effects of heat-shock and aforementioned reagents with much-increased intensity to achieve the goal.

### 3.5. The Optimization of the Heat-Shock Treatment on Nostoc sp.

**Figure 7:**
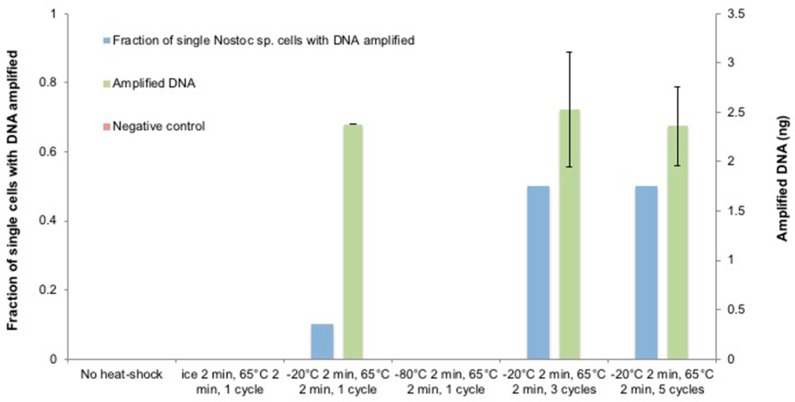
The temperature range effect and cycles of heat-shock on *Nostoc* sp. lysis for SC-WGA. After heat-shock treatment, a mixture of 200 U/µL Ready-Lyse lysozyme and 0.5 mM EDTA was added and incubated at 37 °C for 2 h. The D2 alkaline lysis buffer was then added and incubated at 65 °C for 2 h. *N* = 10 single cells per condition; error bars represent standard deviation.

We started the investigation by treating the single cells with 200 U/µL Ready-Lyse lysozyme after heat-shock (Figure 7). Unlike *C. glutamicum*, a cycle of ice/65 °C heat-shock did not pose any detectable effect on *Nostoc* sp., instead, a cycle of −20 °C/65 °C heat-shock led to 10% of single-cell amplification with 2.38 ng of DNA. However, no amplification was observed when a cycle of −80 °C/65 °C heat-shock was performed. These phenomena show that bacteria with thicker walls are able of enduring more aggressive thermal treatment with a larger temperature difference. Even so, extreme temperatures are not ideal treatments for cell lysis even for species with thick, rigid and multilayered cell walls due to the possibility of DNA shearing during ice crystal formation although the cell walls may be shattered to some extent. To reinforce the *Nostoc* sp. cell wall destabilization while preserving the integrity of the DNA template, a −20 °C/65 °C heat-shock was performed for 3 cycles consecutively. As expected, the single-cell amplification rate was increased to 50% but the average DNA amount still remained low. The further increase in the number of cycles did not induce statistically significant improvement on either the amplification rate or the DNA amount. The tests on both *C. glutamicum* and *Nostoc* sp. cells show that appropriate temperatures and cycles of heat-shock apparently assisted the cell wall destabilization but only to a limited extent. 

### 3.6. The Optimization of Lysozyme Combined with the DTT Treatment on Nostoc sp.

Therefore, chemical effects were investigated to sequentially degrade the multi-layered cyanobacterial cell walls following the heat-shock treatment. Even though lysozyme is known for degrading peptidoglycan layers [75,76] and interfering with nucleic acid polymerase [42,77], for the cyanobacterium, the peptidoglycan layers are encapsulated by the thick external layer and outer membrane that prevents lysozyme to permeate through. Therefore, DTT was used as an agent for breaking disulfide bonds and disrupt polysaccharide of the external layer [70,71,72].

**Figure 8:**
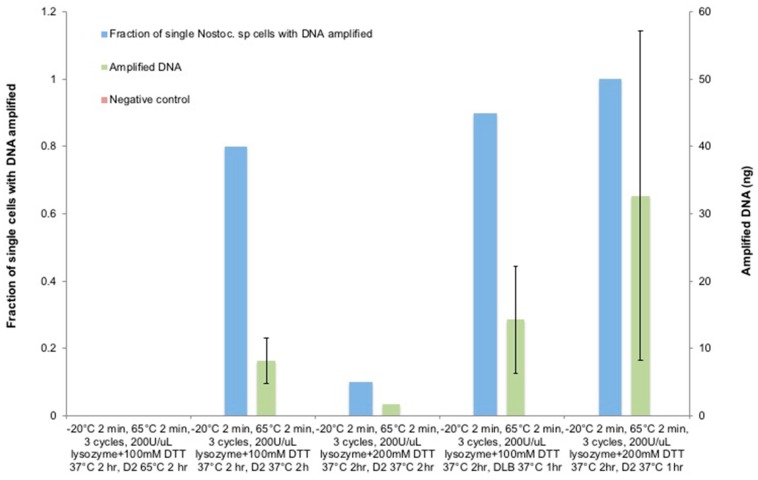
The DTT effect on *Nostoc* sp. lysis for SC-WGA. After the heat-shock treatment, a mixture of 0.5 mM of EDTA, 200 U/µL of Ready-Lyse lysozyme, and the DTT of different concentrations was added to the cells and incubated at 37 °C for 2 h, followed by adding a D2 alkaline lysis buffer. *N* = 10 single cells per condition; error bars represent standard deviation.

Based on the optimal lysis condition investigated to this point, different concentrations of DTT and its incubation conditions were tested following the 3 cycles of −20 °C/65 °C heat-shock treatment and the incubation of a mixture of 100–200 mM DTT, 200 U/µL Ready-Lyse lysozyme, and 0.5 mM EDTA at 37 °C for 2 h (Figure 8). The procedure was followed by the addition of the D2 lysis buffer and incubation at 65 °C for 2 h. However, this attempt did not lead to any detectable amplification. In spite of the thick and complex cyanobacterial cell wall structure, we assume that the elevated incubation temperature of the D2 buffer poses a very narrow window of optimal incubation time which is tedious and time-consuming to pinpoint, and lowering its incubation temperature would broaden the window of optimal incubation length and allow for the timely stopping of the reactions prior to the over-denaturation of the released DNA templates. Therefore, the D2 buffer incubation temperature was decreased to 37 °C while all the other parameters remained unchanged. As expected, the amplified single cell rate ramped to 80% with an average of 8.21 ng of DNA. This suggests that penetrating the external layers of *Nostoc* sp. is critical for lysozyme to reach and digest the peptidoglycan layers. 

As an attempt to further increase the DNA amount, the DTT concentration was doubled to 200 mM; however, that caused only a 10% amplification rate with a minute amount of DNA. Since the increased DTT concentration was expected to degrade the external layer more sufficiently as with *C. glutamicum*, the decline in the amplification rate could be explained by the possibility that the over-denaturation still occurred due to the extended D2 buffer incubation despite the lowered temperature. To test this assumption, we incubated the D2 buffer for 1 h, and the tests were repeated on the use of 100 mM and 200 mM DTT with all the other parameters unaltered. In the test with 100 mM DTT, the reduced incubation time of D2 buffer doubled the amplified DNA amount to 14.32 ng; while in the test with 200 mM DTT, 100% of amplified *Nostoc* sp. single-cell lysis and amplification rate and 32.7 ng of DNA was achieved, and 80% of single-cell replicates reached >25 ng DNA with statistically significant improvements compared to the prior attempt (*p*-value = 0.002). 

Despite of the fact that *C. glutamicum* and *Nostoc* sp. differ greatly in cell wall structures, it is worthwhile to mention that the chemical components of the customized lysis buffer mixture, their concentrations, and incubation temperature necessary to achieve optimal lysis condition for the subsequent DNA amplification turned out to be the same (200 U/µL Ready-Lyse lysozyme, 200 mM DTT, 0.5 mM EDTA, 37 °C). This shows that the customized mixture has the potential to be used without modification for lysing many other bacterial species, while its incubation time is determined by the thickness and the nature of the cell walls. Thermal treatment poses a distinctive effect on species that have thick and rigid cell walls, but may not be necessary for those with comparatively thinner cell walls. As a last step of lysis and denaturing template DNA, we recommend using the Qiagen D2 buffer at 37 °C or lower to pinpoint an optimal incubation time for the customized lysis buffer. 

### 3.7. The Evaluation of the Optimized Lysis Protocol Using Gloeocapsa sp. and Sphaerocystis sp.

**Figure 9:**
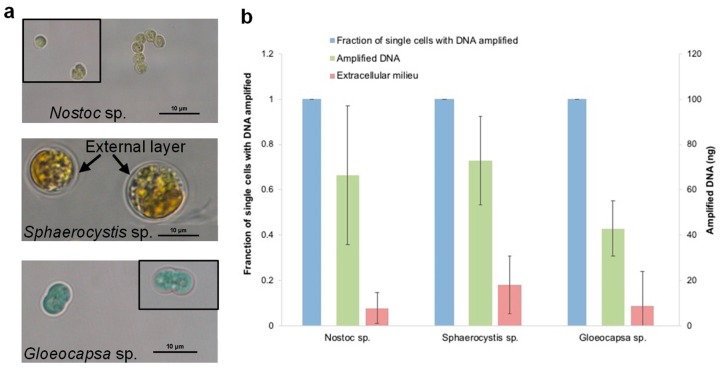
(**a**) Microscopic images of *Nostoc* sp., *Sphaerocystis* sp. and *Gloeocapsa* sp. The black boxes indicate additional cells from another capture to representatively illustrate cells in suspension. (**b**) The use of the developed lysis protocol on *Sphaerocystis* sp. and *Gloeocapsa* sp. for lysis and SC-WGA. *N* = 10 single cells per species per condition; error bars represent standard deviation. Each set of experiment was repeated three times. The sterile PBS as a true negative control did not show any contamination after amplification (not shown in the graph).

**Figure 10:**
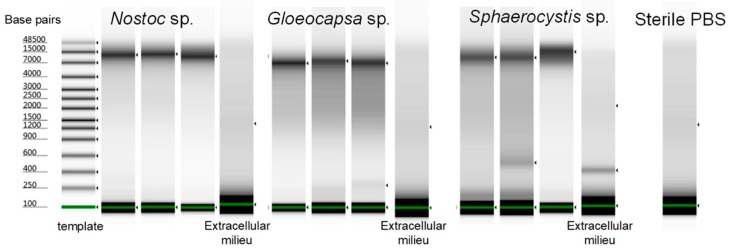
The Tapestation results of amplified DNA from single *Nostoc* sp., *Gloeocapsa* sp. and *Sphaerocystis* sp. cells using the optimized protocol. The DNA was not degraded. Three single cells from each species were shown.

To validate the effectiveness of this bacterial lysis method for especially rigid species in single-cell genomic studies, we tested the optimized buffer mixture and lysis conditions on another two hard-to-lyse species (Figure 9). *Gloeocapsa* sp. was chosen as the first target due to the significant challenges of cell wall disruption, even in bulk studies. *Sphaerocystis* sp. was chosen as a second target because even though it is a eukaryotic algal species, its very thick extracellular matrix, mainly composed of polysaccharides, encapsulated the cells, making it equally hard to penetrate for single-cell DNA amplification in our earlier attempts. Due to these reasons, we assume that these two species would be lysed sufficiently for amplification by directly using the protocol optimized for *Nostoc* sp. without further efforts or modification. Each set of validation experiment was repeated three times for *Nostoc* sp., *Sphaerocystis* sp. and *Gloeocapsa* sp. As expected, the results show that all three species reached a 100% single-cell amplification rate and an average of 66.5 ng, 73.0 ng and 42.8 ng of DNA respectively, and 100% of single-cell replicates amplified to >25 ng DNA. However, *Nostoc* sp., *Gloeocapsa* sp. and *Sphaerocystis* sp. had an average of 7.8 ng, 18.1 ng, and 8.7 ng of DNA in the extracellular milieu that the cells were suspended in, but no DNA was detected in the sterile PBS after amplification. However, the amplified genomic DNA showed a reasonable quality and was easily distinguished from the extracellular milieu based on the Tapestation results (Figure 10). We concluded that there was extracellular DNA in the cell suspension which was most likely caused by the process of dissociating the cell clusters into single-cell suspensions for species that are tightly clustered. For *Nostoc* sp., pestling was necessary to obtain sufficient single cells; and for *Gloeocapsa* sp. and *Sphaerocystis* sp. the dissociation relied on extended pestling and mild sonication, respectively, which could lead to cell disruption. Besides, unlike pure bacterial cultures, the environmental samples would be more likely to contain contaminants.

## 4. Conclusions

Single cell whole genome sequencing has found various applications in mammalian cells due to the ease of cell lysis for genome amplification. However, it has rarely been applied to microbial cells, and one of the major hurdles is the sufficient lysis of the multilayered cell walls without compromising the integrity of the minute amount of DNA template for subsequent amplification. This challenge is especially prominent in microfluidic platforms as the microfluidic-based genome amplification is incompatible with standard bacterial cell lysis methods and commonly used lytic chemicals. This work focused on developing effective bacterial single-cell lysis methods and performed subsequent SC-WGA in a microfluidic platform to obtain >25 ng genomic DNA sufficient for downstream processing. By combining thermal treatment and chemicals including lysozyme, EDTA, DTT and alkaline-based buffer, 100% of the bacterial single-cell lysis rate was achieved for both gram-positive and gram-negative species including *C. glutamicum*, *Nostoc* sp. and *Gloeocapsa* sp., as well as *Sphaerocystis* sp.—a hard-to-lyse eukaryotic species, without resorting to off-chip conventional steps, polymerase-inhibitive reagents or multiple rounds of amplification. Even though species may react differently to the same lysis protocol, a combination of three primary lysis treatments applicable in microfluidic platforms that degrade bacterial cell walls in their distinct ways offers a baseline for adapting the parameters to reach optimal conditions for species of interest. This work addressed one of the major obstacles of applying SC-WGS technologies to the microbial cells by providing a single-cell lysis guideline that can be adapted to facilitate the lysis and amplification of various types of bacterial and algal cells that are challenging for single-cell genome analyses in microfluidic systems. Ultimately, we envision that it would be possible to perform single-cell genomic studies on a vast range of microbial cells in various research including environmental studies and the genomic investigation of rapidly-growing and deadly but multi-drug resistant microbial pathogens such as ESKAPE [78] (*Enterococcus faecium*, *Staphylococcus aureus, Klebsiella pneumoniae, Acinetobacter baumannii, Pseudomonas aeruginosa* and *Enterobacter* spp.) using microfluidic platforms and would potentially lead to the discovery of effective therapies. 

## Supplementary Materials

The following are available online at http://www.mdpi.com/2072-666X/9/8/367/s1.
