# Supplementary material for: The Development of an Effective Bacterial Single-Cell Lysis Method Suitable for Whole Genome Amplification in Microfluidic Platforms
Source: Micromachines (Basel). 2018 Jul 25;9(8):367. doi: 10.3390/mi9080367 (PMC6187716; doi:10.3390/mi9080367)
Supplement: Supplementary file 1 [file micromachines-09-00367-s001.pdf]

Supplemental Materials: Development of Effective Bacterial Single Cell Lysis Method Suitable for Whole Genome Amplification in Microfluidic Platforms

Yuguang Liu, Dirk Schulze-Makuch, Jean-Pierre de Vera, Charles Cockell, Marina Walther-Antonio

1. Methods

1.1.Microfluidic Chip Layout and Experimental Procedure

The PDMS-based microfluidic chip used in the work was illustrated in Figure S1(a). It contains 10 reactions lines for SC-WGA and 2 reaction lines for negative controls. The general on-chip experimental procedure is shown in Figure S1(b). Briefly, samples were introduced into the sample channel and single cells were selected and transported into the cell isolation chambers using laser tweezers. The channel was then flushed with PBS to wash redundant cells out of the chip. Next, reagents were added into the chambers in a sequential manner which include customized lysis buffer (lysozyme and DTT mixture), D2 lysis buffer and neutralization buffer (3.5 nL each). Finally, master mix was added into the amplification chamber, making the total reaction volume 60 nL followed by overnight incubation.

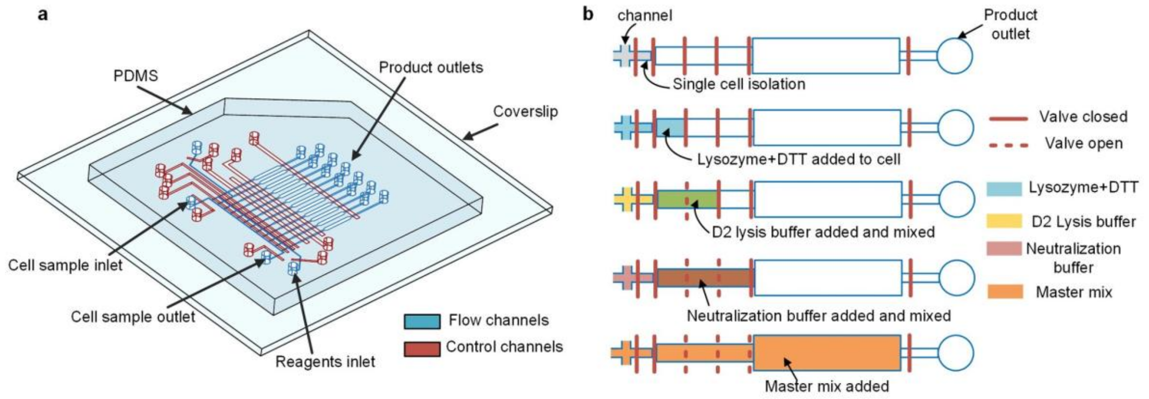

Figure S1. (**a**) Schematic view of a PDMS microfluidic chip for SC-WGA. (**b**) An illustration of on-chip SC-WGA procedure which involves the sequential opening of valves for single cell isolation, addition of lysozyme and DTT mixture, D2 lysis buffer, neutralization buffer and master mix.

2. Mold Fabrication

The photomask for mold fabrication was designed in AutoCAD (Autodesk, USA). SU-8 2025 photoresist (MicroChem, MA, USA) was used to fabricate the mold of the control layer on a silicon wafer, with thickness of 50 µm by spinning the wafer at 3600 rpm for 60 s. The wafer was soft-baked at 65 °C for 2 min and 95 °C for 6 min, and exposed for 16 s at the intensity of 35 mJ/cm^2. After the exposure, the wafer was baked at 65 °C for 2 min and 95 °C for 6 min. After development, the wafer was hard-baked at 150 °C for 30 min. AZ 9260 photoresist (Microchemicals) to fabricate the flow mold, with thickness of 38 µm by two spin coating process. First, hexamethyldisilazane (Sigma Aldrich) was spin-coated onto the silicon wafer at 1200 rpm for 30 s to enhance the adhesion between AZ 9260 and the wafer. A first layer of AZ 9260 was spin-coated at 900 rpm for 80 s, and incubated at 90 °C for 1 min and 110 °C for 4 min. A second layer of AZ 9260 was spin-coated onto the wafer at 900 rpm for 80 s, and incubated at 90 °C for 1 min and 110 °C for 8 min. After development, the wafer was incubated at 120 °C for 2.5 min to allow for the reflow to form the rounded channel profile.

3. PDMS Chip Fabrication

The microfluidic chips were fabricated using multilayer soft lithography. The molds were first treated with sigmacote (Sigma Aldrich) to prevent PDMS from sticking to the photoresist patterns. Both the control layer and the flow layer were made from RTV 615 PDMS (Momentive). PDMS mixture (20:1) was spin-coated on the control mold at 1400 rpm for 60 s, and the mold was set on a leveled surface for 1 hr. The control mold was baked at 80 °C for 10 min and cooled to room temperature. 30 g PDMS mixture (5:1) was poured on the flow mold, and degased and baked at 80 °C for 10 min. Patterned PDMS was peeled off from the flow mold, and inlet and outlet ports were punched manually. The flow layer was manually aligned onto the control layer and incubated 80 °C for 35 min. PDMS was cut and peeled off the control mold and ports were punched in the same manner. 20:1 PDMS mixture was spin-coated on a microscope coverslip at 2000 rpm for 60 s, and baked at 80 °C for 9 min. The chip was bonded to the coverslip and baked at 80 °C overnight. The chip was UV-sterilized for 45 min prior to use.
